# Predominant aspects of knowledge and practical skills among medical students with online learning during the COVID-19 pandemic era

**DOI:** 10.1080/10872981.2023.2182665

**Published:** 2023-02-28

**Authors:** Visuddho Visuddho, David Nugraha, Rezy Ramawan Melbiarta, Rimbun Rimbun, Abdul Khairul Rizki Purba, Irmi Syafa’ah, Arief Bakhtiar, Purwo Sri Rejeki, Achmad Chusnu Romdhoni

**Affiliations:** a Medical Program, Faculty of Medicine, Universitas Airlangga, Surabaya, Indonesia; b Department of Anatomy, Histology, and Pharmacology, Faculty of Medicine, Universitas Airlangga, Surabaya, Indonesia; c Department of Pulmonology and Respiratory Medicine, Faculty of Medicine, Universitas Airlangga/Dr. Soetomo General Hospital, Surabaya, Indonesia; d Department of Medical Physiology and Biochemistry, Faculty of Medicine, Universitas Airlangga, Surabaya, Indonesia; e Department of Otorhinolaryngology-Head & Neck Surgery, Faculty of Medicine, Universitas Airlangga/Dr. Soetomo General Hospital, Surabaya, Indonesia

**Keywords:** Medical skills, online learning, COVID-19, cognitive, education

## Abstract

**Background:**

Social restrictions due to the COVID-19 pandemic have shifted most learning methods into online courses, especially for medical skills education. However, the effects of online courses on medical skill education amongst medical students are still arguable. The study aims to analyse medical students’ knowledge, attitude, practice and satisfaction towards medical skills between online and offline courses.

**Method:**

We performed a case–control study conducted among 533 medical students with online (as a case group, *n* = 288) and offline courses (as a control group, *n* = 245). We evaluated three fundamental medical skills, including history taking [HT], lung physical examination [LPE], and heart physical examination [HPE]. We tested the knowledge and skills among students through theory and practical examinations. Students’ attitudes and satisfaction were assessed using a validated questionnaire.

**Results:**

The scores for knowledge and practical skills among the online group were significantly higher (*p* = 0.016, *p* = 0.004, respectively). In comparison, the scores for the students’ attitudes and satisfaction were substantially lower (*p* = 0.000, *p* = 0.003, respectively) compared to the control group. Most of the students in both groups passed the exam (case vs. control = 81.94%; 83.27%, respectively). Males were the only factor associated with a higher rate of passing the examination (OR 0.42, 95% CI [0.27–0.67], *p* = 0.000).

**Conclusions:**

Online learning could be an alternative approach on improving student’s knowledge and practice towards medical skill especially amidst COVID-19 pandemic, however further consideration on student’s attitude and satisfaction are mandatory to achieve appropriate competence as future general practitioner.

## Introduction

Since the World Health Organisation (WHO) has declared COVID-19 as a global pandemic, COVID-19 cases have continued to increase, and even some countries are facing a new wave of the pandemic. All countries in the world are still struggling to combat COVID-19, and not a single country has succeeded yet in conquering it completely [1]. As of the end of 2021, the WHO reported more than 254 million confirmed cases, with more than 5 million deaths and affected more than 221 countries or territories [2]. Indonesia is also struggling with this pandemic, and the government has attempted various policies such as large-scale social restrictions, strict stay-at-home orders, and increased health-care services across the country [3]. Recently, although many efforts have been made to break the chain of transmission, there is no sign that the pandemic will end, and no one can guarantee that the cases will not increase anymore.

This pandemic had a tremendous impact on human life, not only physical health but also almost all aspects of life have shifted to the virtual world, including medical education [4]. It is undeniable that the learning system in all medical faculties in low–middle-income countries, such as Indonesia, has changed completely. In a very short period of time, medical faculties are required to adapt to implement virtual teaching and learning processes. Virtual learning offers many benefits not found in conventional systems, such as broad accessibility from anywhere and anytime, asynchronous discussions with classmates, immediate feedback on tests, and flexibility [5]. However, despite the benefits of virtual learning, its implementation is very challenging, especially in medical schools with various complex learning processes.

Medical education always involves three interrelated aspects of values, namely, cognitive, affective, and psychomotor. Medical education often requires adequate clinical exposure and practice; however, because the risk of being infected is much greater for face-to-face learning, online education is the best option at this time [6]. On the other hand, in principle, the ability of medical skills cannot be completely replaced virtually. Consequently, reduced exposure to the direct application of medical skills will ultimately reduce students’ performance in conducting examinations, students’ confidence, and students’ overall abilities as doctors in the future [6]. Previous research reported that the disadvantages of virtual teaching in medical education include technical difficulties, reduced active student involvement, and the loss of some aspects of assessment [7].

Apart from the unprecedented pandemic conditions, the medical faculty has a role in preparing and ensuring that its students continue to acquire competencies in accordance with the learning targets. Previous studies have revealed that virtual learning may hinder and limit students to cultivate necessary skills due to the lack of hands-on practices [8,9]. This may have serious implications for students who might face difficulties in clinical settings, resulting in lower confidences performances. Therefore, this study aims to evaluate the impact of virtual medical skills training on students’ knowledge, attitude, and practice, as well as their satisfaction with virtual learning. In addition, this research also serves as an evaluation of the methods used in teaching and learning process. We expected to provide information that can be useful to formulate learning policies during the COVID-19 pandemic.

## Method

### Study design and participants

This study was a case–control study of university students enrolled at the Faculty of Medicine, Universitas Airlangga (Surabaya, East Java Province, Indonesia). The Faculty of Medicine Universitas Airlangga is one of the best medical faculties and has become the second oldest medical school in Indonesia. The case group was the student given the online learning for medical skills training, while the control group was the student given the offline learning for medical skills training. Participants who had participated in the medical skills training were included in this study. Participants who did not adhere to medical skills training, did not participate in the examination, did not fill out the questionnaire, or wished to refuse during the study were excluded.

The participants were given three medical skills subjects, (1) history taking (HT), (2) heart physical examination (HPE), and (3) lung physical examination (LPE). For each medical skill subject, one session of theory lecture and two sessions of skill demonstration and practice were given online for the case group and offline for the control group. Each session was conducted in 2 hours with professional and certified lecturers. The study has been approved by the Research Ethics Commission of the Faculty of Medicine Universitas Airlangga (No.147/EC/KEPK/FKUA/2021) and conducted following the Helsinki Declaration.

### Theory examination score

Predominant aspects of knowledge and practical skills among medical students with online learning during the COVID-19 Knowledge assessment was obtained from the scores of the participants during the theory examination. The theory examination was conducted after the participants finished all courses. A total of four multiple-choice questions for each subject were given to participants. The questions provided by lecturers based on the course materials. One correct answer was marked as 25 points, while the empty or wrong answers were marked as zero points. Therefore, there will be a maximum of 100 points for each subject. We calculated the score for each subject and average of three subjects.

**Figure 1.**
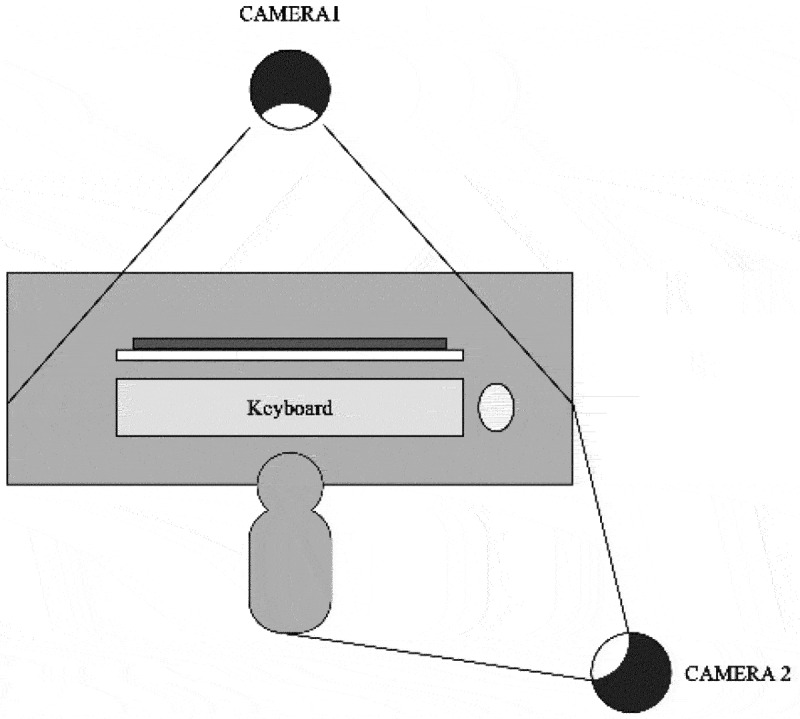
Online examination supervision.

For the case group, the theory examination was conducted online using an online computer-based test, while for the control group, a computer-based test was conducted offline in the examination room. To avoid cheating during an online examination, we strict the rules by using the safety exam browser application (safeexambrowser.org), which can restrict the participants to open other applications during online examination. The online examination was also supervised by a video conference meeting (zoom.us), and students were asked to keep their eyes straight to the laptop, both hands appear in the video, no other person in their room, and strictly observed from a zoom camera as demonstrated in Figure 1. We also made a different question for two groups since the examination was conducted at different times.

In addition, we analyzed the difficulty index and discrimination index to assess whether there were any differences in the level of difficulties between the online and offline knowledge examination questions. We calculated both the difficulty index and the discrimination index using a formula based on previously published research [8]. The difficulty index was calculated by dividing the total correct answers by the total respondents. The difficulty index was categorised as very difficult (0-0.19), difficult (0.2-0.29), moderate (0.3–0.69), easy (0.70-0.85), and very easy (>0.85). Subsequently, the discrimination index was calculated by dividing the difference of responses between correct and incorrect answers to the half of the participants tried to answer. The discrimination index was classified as poor (0.00–0.19), marginal (0.2–0.29), good (0.3–0.39), and very good (≥0.4) [9].

### Skill examination score

The evaluation of practice was assessed using the scores of participants during the skill examination. The participants performed a total of 10 step-by-step procedures within 6 minutes during the skill examination. For each step, 10 points were given for participants doing the step precisely, 5 points for participants doing the step not accurately, and 0 points for the participants not doing the step; therefore, a maximum of 100 points was given if participants fulfilled all the procedures precisely. Certified lecturers assess the procedures via an online video conference (using *zoom.us)* for the case group and offline in the skill test room for the control group.

To investigate the bias rise from different skill examination methods between the two groups, we constructed a questionnaire for the examiner to evaluate the objectivity in skill examination assessments. This questionnaire adopted several domains from the Objective Structured Clinical Examination (OSCE) objectivity assessment, namely, Halo Effect (statement number 1–3), Understanding (statement number 4–5), and Agreement (statement number 6–8) that reform to several signalling statements. All statements displayed as Likert scale. The examiner filled in the questionnaire from strongly disagree (score 1) to strongly agree (score 5) to each statement.

Before the questionnaire is distributed to the examiner, the questionnaire has been validated by six experts of OSCE assessor using the Content Validity Index (CVI). The Item-CVI for each question is higher than 0.83, indicating a validated item. The Scale-CVI of the whole questionnaire is 0.98, determined as a validated questionnaire. The questionnaire also has been validated statistically using the bivariate correlations test and showed a significant result.

### Attitude and satisfaction score

Because it was not feasible to do an interview during COVID-19 social distancing, we used the online questionnaire (using *form.google.com*) to measure the students’ attitude and satisfaction. This form contained a brief introduction about the background, aims, procedures, information for consent, and informed consent. Participants in each group filled out the form guided by the researcher in the video conference application (using *zoom.us*) to avoid misperception of the questionnaire. The questionnaire was constructed from several attitude domains, including willingness, understanding, capacity, application, intended behaviour, and self-efficacy, which can be seen in **Supplementary Table S1**.

A preliminary validation was conducted with the expert of Indonesian language and expert of medical skill education to enhance the comprehension of the questionnaire. We then conduct a survey within 41 preliminary samples and statistically tested using bivariate correlation test and reliability test. The questions were valid with the Cronbach’s alpha coefficient 0.794 indicating good internal reliability. There were six statements on the questionnaire that were displayed on the Likert scale (1–5). The total score for each participant was obtained by the accumulation of the Likert scale (maximum 30 points).

Participants state their satisfaction on several aspects of the course, including instructor, preparation, effort, facilities, learning goals, and learning method after the course using the satisfaction questionnaire. The preliminary survey was conducted together with the attitude questionnaire to ensure the quality of the questionnaire. There were six statements on the questionnaire that were displayed on the Likert scale (1–5). The total score for each participant was obtained by the accumulation of the Likert scale (maximum 30 points).

### Data collection

We collected data of participants’ age, gender, admission, district zone, theory examination score, skill examination score, and questionnaire reports. The theory and skill examination scores were collected in the examination database of the Faculty of Medicine, Universitas Airlangga. We analysed the pass criteria for examinations with minimum scores of 65 for average theory examination and minimum 70 for the skill examination of each subject, as defined by the Curriculum Board of Faculty of Medicine, Universitas Airlangga. The attitude and satisfaction questionnaires were distributed using an online questionnaire form (*form.google.com*). To neutralize observer bias, we use the student number instead of name in filling the data to avoid duplicate data. The data privacy was declared during a video conference meeting (zoom.us) with all participants. The objectivity examiner questionnaire was also distributed using an online questionnaire form (*form.google.com*).

### Statistical analysis

The Mann–Whitney U-test and The Chi-square test were used to compare differences between the online and offline groups. Levene’s test was done to identify the equality of variances. Logistic regression was performed to explore the factors that likely affect the pass of the exam. Statistically significant was considered using two-sided α with a p-value less than 0.05. All statistical analyses were performed using the IBM SPSS software (version 13).

## Results

### Study participants characteristics

**Figure 2.**
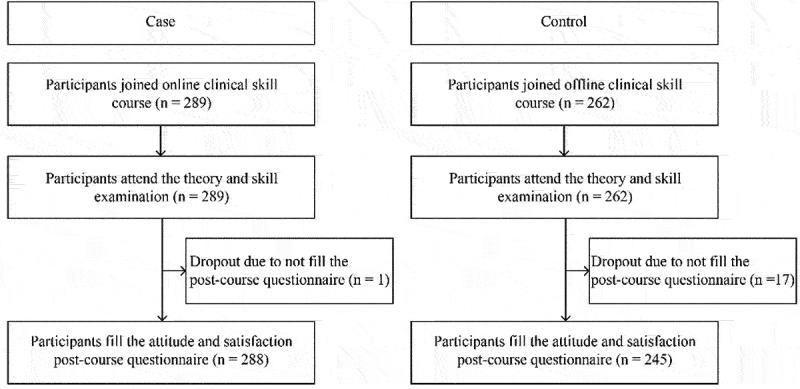
Study flowchart.

**Figure 3.**
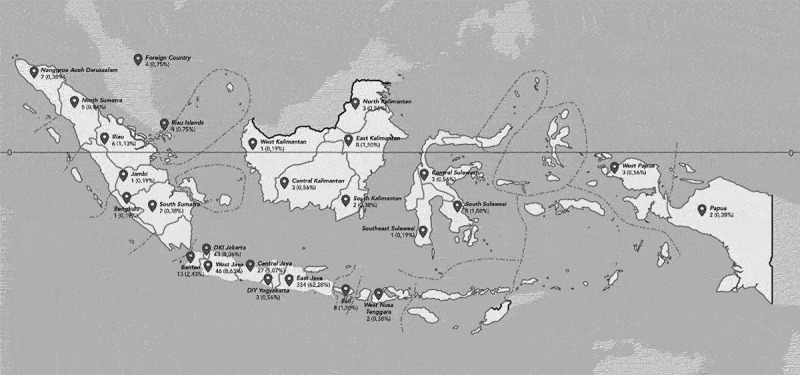
Demographic distribution of participants.

The study flow chart is demonstrated in Figure 2. The assessment was done on 288 students from the online group and 245 students from the offline group. The majority of the participants were female in both online (63.19%) and offline (63.27%) groups as shown in Table 1. The demographic distribution is shown in Figure 3. The students were from different areas from all over Indonesia. Participants in the online group were significantly older when compared to those offline (*p* < 0.001). They were accepted to the university from the same path with a similar proportion; the highest proportion was from the national test path (online: 31.25%; offline: 37.55%), and the least one was from the international program (online: 16.60%; offline: 7.35%). There was no difference between groups on the exam pass rate (online 81.94% vs offline 83.27%; *p* = 0.848).

**Table 1.** Baseline demography of study participants.

Variables	Online Group (288)	Offline Group (245)	Comparation	Levene’s Test	
			p-value	F	*p*-value
Age. median (IQR)	19.00 (18.00–19.00)	19.00 (19.00–20.00)	0.000*	0.267	0.605
Gender (Female)	182 (63.19%)	155 (63.27%)	0.947	-	-
Admission path			0.011	-	-
- National Invitation	72 (25.00%)	68 (27.76%)	0.471	-	-
- National Test	90 (31.25%)	92 (37.55%)	0.126	-	-
- Independent Path	77 (26.74%)	67 (27.35%)	0.946	-	-
- International Program	48 (16.67%)	18 (7.35%)	0.001*	-	-
Exam pass rate	236 (81.94%)	204 (83.27%)	0.848	3.121	0.078

*p < 0.05 is considered statistically significant.

### Knowledge, attitude, practice, and satisfaction between groups

The comparison of knowledge, attitude, and satisfaction between groups is shown in Table 2. In the HT and HPE subjects, the knowledge score was higher in the offline group when compared to online, although a significant difference was found only in the HPE subject (*p* = 0.013). In contrast, the knowledge score of the LPE subject was significantly higher in the online group (*p* < 0.001). On the other hand, practice scores of the HT and HPE subjects were significantly higher in the online group (HT: *p* = 0.025; HPE: *p* = 0.001); whilst in the LPE subject, the contrast was shown (*p* = 0.035). However, the total knowledge and practice score was significantly higher in the online group (knowledge: *p* = 0.016; practice: *p* = 0.004).

**Table 2.** Comparison of knowledge, attitude, practice, and satisfaction between online and offline groups.

Variables	Online (n=288)		Offline (n=245)		*p*-value
	Mean Rank	Median (IQR)	Mean Rank	Median (IQR)	
Knowledge	281.47	83.33 (66.67–83.33)	249.99	75.00 (66.67–83.33)	0.016*
- HT	257.00	75.00 (50.00–75.00)	278.75	75.00 (75.00–75.00)	0.059
- LPE	312.31	100.00 (75.00–100.00)	213.74	75.00 (50.00–100.00)	0.000*
- HPE	252.83	75.00 (75.00–100.00)	283.66	75.00 (75.00–100.00)	0.013*
Practice	284.46	96.67 (93.33–100.00)	246.48	95.00 (91.67–98.33)	0.004*
- HT	280.09	95.00 (90.00–100.00)	251.61	95.00 (90.00–100.00)	0.025*
- LPE	255.51	100.00 (90.00–100.00)	280.51	100.00 (95.00–100.00)	0.035*
- HPE	285.10	100.00 (95.00–100.00)	245.73	100.00 (90.00–100.00)	0.001*
Attitude	203.52	22.00 (19.75–24.00)	341.62	25.33 (22.83–27.83)	0.000*
- HT	213.84	23.00 (20.00–25.00)	329.49	25.00 (23.00–28.00)	0.000*
- LPE	202.84	22.00 (19.00–24.00)	342.42	25.00 (23.00–28.00)	0.000*
- HPE	208.21	22.00 (19.00–24.00)	336.11	25.00 (23.00–28.00)	0.000*
Satisfaction	175.00	20.00 (17.00–22.00)	375.15	26.00 (23.00–28.50)	0.000*

*p < 0.05 is considered statistically significant. HT: history taking; LPE: lung physical examination; HPE: heart physical examination.

The students’ attitude was higher in the offline group compared to the online. The pattern was similar when the subject assessed the attitude separately. Significant differences were found in HT, LPE, HPE, and the whole attitude between groups (*p* < 0.001). Participants’ satisfaction was also significantly higher in the offline group than in the online group (*p* < 0.001).

### Assessment of item difficulty and discrimination index

Question items were analyzed to assess difficulty and discrimination index, as shown in Table 3. Overall subject’s difficulties were categorised as easy in both groups. The HT subject was considered of moderate difficulty in the online group, while the remaining was easy, while all of the subjects in the offline group were easy. In the discrimination index variable, all of the subjects were good at discriminating students’ abilities both in the online and offline groups.

**Table 3.** Difficulty and discrimination index.

Items	Difficulty index		Discrimination index	
	Index	Interpretation	Index	Interpretation
Online group				
HT	0.676	Moderate	0.243	Marginal
LPE	0.837	Easy	0.301	Good
HPE	0.756	Easy	0.417	Very good
Mean	0.756	Easy	0.321	Good
Offline group				
HT	0.701	Easy	0.232	Marginal
LPE	0.701	Easy	0.458	Very good
HPE	0.796	Easy	0.247	Marginal
Mean	0.732	Easy	0.312	Good

HT: history taking; LPE: lung physical examination; HPE: heart physical examination. Difficulty index >0.85 very easy; 0.70-0.85 easy; 0.30–0.69 moderate; 0.2-0.29 difficult; 0-0.19 very difficult. Discrimination index ≥0.40 very good; 0.30–0.39 good; 0.20–0.29 marginal; 0.00–0.19 poor; <0.00 rejected.

### Examiner objectivity questionnaire

We obtained a total of 96 responses by the skill examiner (46 online and 50 offline). Most of them were not affected by students’ personality characteristics (self-confidence, first-impression, and communication skills; *p* = 0.276, *p* = 0.329, *p* = 0.264, respectively). The instruction was clear, and the examiner understood it (*p* = 0.238; *p* = 0.061). Students’ skills were examined objectively based on their real-time skills (*p* = 0.071). This was shown by insignificant differences found when comparing the objectivity between online and offline examiners, as presented in Table 4. However, the offline group were significantly more adhered to operational definitions and had appropriate learning objectives (*p* = 0.021, *p* = 0.011, respectively).

**Table 4.** Comparison of objectivity of examiner between two assessment methods.

Statement	Median (IQR)		*p*-value
	Online (n=288)	Offline (n=245)	
Not affected by participants self-confidence	2.00 (2.00–3.00)	2.00 (2.00–3.00)	0.276
Not affected by first-impression	2.50 (2.00–4.00)	2.00 (2.00–4.00)	0.329
Not affected by communication skills	2.00 (2.00–2.00)	2.00 (1.00–2.00)	0.264
Receive clear instruction	4.00 (4.00–5.00)	4.00 (4.00–5.00)	0.238
Instruction understanding	4.00 (4.00–5.00)	5.00 (4.00–5.00)	0.061
Appropriate learning objective	4.00 (4.00–5.00)	4.00 (4.00–5.00)	0.011*
Adherence to operational definitions	4.00 (4.00–5.00)	5.00 (4.00–5.00)	0.021*
Assess the skills, not the theory of participants	4.00 (4.00–5.00)	4.00 (4.00–5.00)	0.071

*p < 0.05 is considered statistically significant; IQR: interquartile range.

### Analysis factor of passing the examination

Our bivariate logistic regression is shown in Table 5. The probability of male participants passing the exam was significantly lowered by 58% than female participants. The course group (online or offline) does not influence the rate of passing the examination. The satisfaction median also did not significantly affect the rate of passing examinations, however it was higher among students who did not pass the exam. The multivariate analysis shows the significant odds in gender variables. This indicates that gender may be the independent variables in determining the pass of the examination.

**Table 5.** Analysis factor of student exam pass rate.

Factors	Comparative Analysis			Univariate Logistic Regression		
	Pass (442)	Not Pass (91)	*p*-value	β	OR (95% CI)	*p*-value
Age Median (IQR)	19.00 (19.00–19.00)	19.00 (19.00–19.00)	0.959	0.027	1.03 (0.79–1.34)	0.841
Group						
- Online	238 (53.85%)	50 (54.95%)	0.848	Reff		
- Offline	204 (46.15%)	41 (45.05%)		0.044	1.05 (0.66–1.65)	0.848
Sex						
- Female	296 (66.97%)	42 (46.15%)	0.000*	Reff		
- Male	146 (33.03%)	49 (53.85%)		−0.861	0.42 (0.27–0.67)	0.000*
Group-Sex Interaction	-	-	-	−0.562	0.57 (0.33–0.98)	0.043*
Admission Path						
- National Invitation	123 (27.83%)	17 (18.68%)	0.211	Reff		
- National Test	144 (32.58%)	38 (41.76%)		−0.395	0.67 (0.43–1.07)	0.094
- Independent Path	119 (26.92%)	26 (28.57%)		−0.082	0.92 (0.56–1.52)	0.748
- International	56 (12.67%)	10 (10.99%)		0.161	1.18 (0.58–2.4)	0.658
Attitude Median (IQR)	23.33 (20.92–25.67)	23.67 (20.33–26.33)	0.538	−0.015	0.99 (0.93–1.05)	0.613
Satisfaction Median (IQR)	22.00 (20.00–23.00)	22.00 (20.00–24.00)	0.159	−0.066	0.94 (0.87–1.01)	0.081

*p < 0.05 is considered statistically significant.

## Discussion

Medical skills education is one of the keys in the good implementation of medical practice in the future [10,11]. Therefore, it is essential to find an appropriate learning method for students, especially during the COVID-19 pandemic [12]. Our study found significant differences in knowledge, attitude, practice, and satisfaction between participants’ online and offline learning methods. However, we did not find any effect of learning methods on student exam pass rate.

The total knowledge score was significantly higher for online participants than for offline participants. This result linear with a meta-analysis that showed the online learning group having higher post-test scores (SMD = 0.81; 95% CI 0.43–1.20) [13]. Intriguingly, we found that HT and HPE have substantially higher scores in offline groups, yet the LPE still conforms with the main results. The higher knowledge score on offline history taking subject may be explained by discrimination and difficulty index analysis. Our analysis showed that the online group has moderately difficult questions for HT subject, while the offline group has easy questions for HT subject. However, our diverse findings between medical subjects are similar to the Kim et al. study, which reported that several online medical subjects tend to have higher scores, while the other medical subjects tend to have lower scores [14]. They conclude that different subjects will have different preferred methods to give more benefit to the knowledge score [14].

A higher knowledge score is associated with higher retention, which is affected by the student’s self-discipline in learning [15,16]. Self-learning is one of the essential concerns in online education, which can be affected by online learning [17,18]. One study showed that among participants of online learning, the self-learning level is still moderate [17]. Time efficiency obtained during online learning can be used as an opportunity to increase student self-learning. Nevertheless, since self-learning is challenging for many students, further understanding in the way of making e-learning effective was required [19].

The proficiency of a medical student in performing medical skills is crucial and fundamental concerning being a competent practitioner in the future. Before the pandemic starts, the medical skills education is preferred to be done offline, because the simulation is considered to improve the medical practical performance [10,20]. The simulation is reliable for assessing medical skills topics’ learners [21], being a more engaging method to study medical skills [22]. Offline methods reduce technical problems on doing the simulation study [23,24]. However, during this unprecedented teaching restriction in the midst of a pandemic, it is imperative to fulfil their competency and continue their education. Transforming into virtual medical skills lecture seems to be the most suitable approach in this pandemic.

Based on the results of our study, we found that online groups significantly gained higher scores for HT and HPE. Similar to our findings, a prior study assessing virtual methods using virtual patients for HT training showed improvements in global performance, interviewing technique, and logical sequence [25]. In line with this study, another report also revealed that virtual HT significantly increased students’ confidence to two-thirds of nearly half of the respondents [26]. Moreover, a previous study also showed that virtual auscultation courses using interactive case-based webinars were practical, interactive, and well-received by the students [27].

The lung examination skill score was better in the offline group compared to the online group [28]. This examination is performed in the wider area of palpation, percussion, and auscultation, including the chest and the back. Therefore, this examination takes more time compared to HT and HPE. If there are any technical problems in the given 6 minutes, physical examination would be difficult to carry out properly thus reducing student’s performance during skill examination. Moreover, hands-on practice may benefit the students by providing them with the opportunity to learn from their mistakes and observe directly from the tutor’s simulation yet at the same time get instant feedback and correction from the tutor [29]. This will enrich their experiences and translate their practical knowledge into the actual setting, thus providing better outcomes for their performances. This might be the main reason for challenges in performing online LPE skill examinations and why their offline practice scores are higher.

The attitude was described as the learner’s rate of their role when dealing with an issue subjected to the modules, as a health-care professional [30], composed of self-efficacy, willingness, and behaviour domain in the current study. These domains would contribute to developing students’ medical skills and knowledge, such as communication skills and research awareness [31,32]. However, attitude differences between online and offline learning groups are still obvious, considering sudden shifting in teaching methods due to the pandemic.

In the current study, we found that students’ attitudes were significantly lower in the online group of HT, lung, and heart PE subjects when compared to the offline group. A study by Muflih et al. showed that medical students’ attitudes towards online learning were overwhelmingly bad, similar to Coman et al., which these studies support current findings [23,33]. Sudden changes in learning methods have been related to high students’ perceived barriers to online learning. These are lack of learning facility, environment motivation, instructions, and internet access [23,34]. A previous study reported students’ perception who received online teaching experienced more difficulty in understanding classes. This could be related to different socioeconomic burdens during the pandemic and previous experience with online facilities [23]. Other living areas could also limit internet access, considering participants in this study were spread all over the country. This could spend more mental effort with no significant information acquired on the related modules, thus affecting students’ self-efficacy [35]. It was also found that self-efficacy correlates to students’ knowledge [36]. To date, most medical students still prefer face-to-face learning in order to receive higher clinical experience [23].

Virtual learning may provide advantages that cannot be obtained through conventional learning processes. Virtual clinical skills education might provide a detailed approach that medical students could benefit from [37,38]. For example, they could easily record and re-watch the pre-recorded video whenever and wherever they want. At the same time, it helps students visualise the examination procedure. Moreover, the online learning method provides the usage of audiovisual in combination with graphic animation, which allows explaining difficult subjects in a comprehensive way. Course content could also be updated periodically, especially for medical fields where knowledge updates quickly evolved [30,39,40]. This explains why the online tutorial video was rated to be more effective for preclinical students due to its aesthetic, well-organised, and short period than live video [41]. A previous study also reported that more than half of the students found that online teaching was more interactive, with a direct opportunity to ask the lecturer [24]. Small group discussion group presentation and case simulation using quizzes, polling, and breakout rooms seem exciting and successfully increase their engagement during lectures. Online learning also has several advantages, i.e., location flexibility, convenience, cost-saving, and ease of access, which may be acceptable for some people [34,35]. A previous study showed medical students perceived that the online learning method was beneficial [30].

However, the virtual learning process, especially for medical skills training, is not that simple and beneficial yet facing various challenges. It is impossible to gain impeccable experiences, and it seems unrealistic to permanently switch to a virtual method [37]. A nationwide study conducted in 31 medical schools in the United Kingdom showed more than 75% of the medical students felt that virtual learning was unsuccessful in replacing the direct face-to-face method [24,42]. Moreover, most medical students said they could not learn practical clinical skills through a virtual teaching approach. Similarly, another study concerned that virtual teaching might compromise medical students’ competency in the clinical setting [43]. It is worth to note that students might also feel unprepared for their future profession due to virtual learning, especially in their clinical skills ability [44]. The lack of the standardised mannequin or patient model, active engagement, and direct contact with the patients made the students more challenging to learn and understand the critical concept of medical skills [37].

Interestingly, our results showed no significant difference in exam pass rate between the students of online and offline groups. We found a small negative relationship between the attitude and satisfaction score with the exam pass rate, but not significant. These findings were contrary to the other studies, in which they showed learning attitude and satisfaction were positively correlated with their performance [45,46]. After an additional analysis, we found no correlation between the attitude or satisfaction score with the exam pass rate (*R* = −0.042; *p* = 0.330 and R = −0.038; *p* = 0.382, respectively). The higher attitude or satisfaction of students do not conclude that they will pay more special effort for examinations. However, this data cannot be interpreted easily, since it may be caused by our exam pass criteria that only used knowledge and practice score. Further study considering the factors of student effort to accomplish the examination is needed to analyse the correlation between student’s perception and academic performance.

Finally, the current evidence still does not show which methods work better for medical education [13]. The best method must be considered depending on the learning goals [47]. Other factors that may involve these results are the participants’ activity during the course [48,49]. If the institution can still maintain the student obligation to be active in the course, the online courses can still be comparable to offline courses. Therefore, for some reasons, online courses can still be implemented as a substitute for offline courses during the COVID-19 pandemic [4].

## Limitation

This study analyses the variables in pre- and post-intervention only, rather than following students’ development across the period of the study. Students’ preferences and perceptions must be considered to seek different effects of learning methods between subjects. Hence, further studies analysing these variables between learning methods, in more subjects using larger samples, are recommended to seek any existing pattern or determining factors. However, a multi-centered institution study is required to give better data on the representation of general medical students across a region since population heterogeneity could not be denied.

## Conclusion

In conclusion, online learning could be an alternative approach to improving student’s knowledge and practice towards medical skills, especially amidst COVID-19 pandemic. Knowledge and practical skills of medical students during the COVID-19 pandemic are higher in online learning. However, further consideration on a student’s attitude and satisfaction is mandatory to achieve appropriate competence as a future general practitioner.

## Supplementary Material

Supplemental Material
