# Supplementary material for: Predominant aspects of knowledge and practical skills among medical students with online learning during the COVID-19 pandemic era
Source: Med Educ Online. 2023 Feb 28;28(1):2182665. doi: 10.1080/10872981.2023.2182665 (PMC9980156; doi:10.1080/10872981.2023.2182665)
Supplement: Supplemental Material [file ZMEO_A_2182665_SM3010.docx]

**SUPPLEMENTAL MATERIAL**

**Predominant aspects of knowledge and practical skills among medical students with online learning during the COVID-19 pandemic era**

Visuddho Visuddho^a^, David Nugraha^a^, Rezy Ramawan Melbiarta^a^, Rimbun Rimbun^b*^, Abdul Khairul Rizki Purba^b^, Irmi Syafa’ah^c^, Arief Bakhtiar^c^, Purwo Sri Rejeki^d^, Achmad Chusnu Romdhoni^e^

*^a^Medical Program, Faculty of Medicine, Universitas Airlangga, Surabaya, Indonesia; ^b^Department of Anatomy, Histology, and Pharmacology, Faculty of Medicine, Universitas Airlangga, Surabaya, Indonesia; ^c^Department of Pulmonology and Respiratory Medicine, Faculty of Medicine, Universitas Airlangga/Dr. Soetomo General Hospital, Surabaya, Indonesia; ^d^Department of Medical Physiology and Biochemistry, Faculty of Medicine, Universitas Airlangga, Surabaya, Indonesia; ^e^Department of Otorhinolaryngology-Head & Neck Surgery, Faculty of Medicine, Universitas Airlangga/Dr. Soetomo General Hospital, Surabaya, Indonesia*

^*^Corresponding author

Email: [rimbun@fk.unair.ac.id](mailto:rimbun@fk.unair.ac.id)

**Supplementary Table 1**.

Questionnaire for Attitude and Satisfaction

| Statement | Offline | | | | | Online | | | | |
| --- | --- | --- | --- | --- | --- | --- | --- | --- | --- | --- |
|  | 1 | 2 | 3 | 4 | 5 | 1 | 2 | 3 | 4 | 5 |
| ATTITUDE OF HISTORY TAKING SUBJECT | | | | | | | | | | |
| Willingness |  |  |  |  |  |  |  |  |  |  |
| I desire to self-learning about history taking technique in patient with shortness of breath. | 0 (0) | 3 (1%) | 35 (14%) | 98 (0,4) | 109 (44%) | 2 (0%) | 28 (9%) | 85 (29%) | 123 (42%) | 50 (17%) |
| Understanding |  |  |  |  |  |  |  |  |  |  |
| I understand the history taking technique in patient with shortness of breath. | 0 (%) | 7 (2%) | 54 (22%) | 109 (44%) | 75 (30%) | 6 (2%) | 32 (11%) | 93 (32%) | 120 (41%) | 37 (12%) |
| Capacity |  |  |  |  |  |  |  |  |  |  |
| I am able to do history taking in patient with shortness of breath. | 0 (0) | 0 (0) | 24 (9%) | 95 (38%) | 126 (51%) | 5 (1%) | 14 (4%) | 54 (18%) | 147 (51%) | 68 (23%) |
| Application |  |  |  |  |  |  |  |  |  |  |
| I am able to apply the history taking technique in patient with shortness of breath | 0 (0) | 1 (0%) | 26 (10%) | 126 (51%) | 92 (37%) | 2 (0%) | 15 (5%) | 60 (20%) | 158 (54%) | 53 (18%) |
| Intended Behaviour |  |  |  |  |  |  |  |  |  |  |
| With my competence, I will do history taking when i meet patient with shortness of breath | 0 (0) | 6 (2%) | 53 (21%) | 101 (41%) | 85 (34%) | 5 (1%) | 19 (6%) | 81 (28%) | 133 (46%) | 50 (17%) |
| Self-Efficacy |  |  |  |  |  |  |  |  |  |  |
| I able to achieve the history taking technique competence according to learning objective | 0 (0) | 2 (0%) | 30 (12%) | 118 (48%) | 95 (38%) | 4 (1%) | 14 (4%) | 74 (25%) | 157 (54%) | 39 (13%) |
| ATTITUDE OF LUNG PHYSICAL EXAMINATION SUBJECT | | | | | | | | | | |
| Willingness |  |  |  |  |  |  |  |  |  |  |
| I desire to self-learning about lung physical examination technique | 0 (0) | 8 (3%) | 29 (11%) | 100 (40%) | 108 (44%) | 4 (1%) | 21 (7%) | 60 (20%) | 122 (42%) | 81 (28%) |
| Understanding |  |  |  |  |  |  |  |  |  |  |
| I understand the lung physical examination technique | 1 (0%) | 10 (4%) | 63 (25%) | 96 (39%) | 75 (30%) | 9 (3%) | 39 (13%) | 97 (33%) | 117 (40%) | 26 (9%) |
| Capacity |  |  |  |  |  |  |  |  |  |  |
| I am able to do lung physical examination correctly | 0 (0) | 0 (0) | 19 (7%) | 98 (0,4) | 128 (52%) | 2 (0%) | 26 (9%) | 66 (22%) | 140 (48%) | 54 (18%) |
| Application |  |  |  |  |  |  |  |  |  |  |
| I am able to interpret the lung physical examination result | 0 (0) | 4 (1%) | 40 (16%) | 108 (44%) | 93 (37%) | 6 (2%) | 41 (14%) | 107 (37%) | 107 (37%) | 27 (9%) |
| Intended Behaviour |  |  |  |  |  |  |  |  |  |  |
| With my competence, I will do lung physical examination when i meet the patient | 0 (0) | 10 (4%) | 53 (21%) | 103 (42%) | 79 (32%) | 8 (2%) | 34 (11%) | 102 (35%) | 119 (41%) | 25 (8%) |
| Self-Efficacy |  |  |  |  |  |  |  |  |  |  |
| I able to achieve the lung physical examination competence according to learning objective | 0 (0) | 2 (0%) | 35 (14%) | 97 (39%) | 111 (45%) | 7 (2%) | 28 (9%) | 100 (34%) | 124 (43%) | 29 (10%) |
| ATTITUDE OF HEART PHYSICAL EXAMINATION SUBJECT | | | | | | | | | | |
| Willingness |  |  |  |  |  |  |  |  |  |  |
| I desire to self-learning about heart physical examination technique | 0 (0) | 6 (2%) | 34 (13%) | 101 (41%) | 104 (42%) | 2 (0%) | 18 (6%) | 65 (22%) | 121 (42%) | 82 (28%) |
| Understanding |  |  |  |  |  |  |  |  |  |  |
| I understand the heart physical examination technique | 3 (1%) | 7 (2%) | 58 (23%) | 104 (42%) | 73 (29%) | 5 (1%) | 36 (12%) | 98 (34%) | 119 (41%) | 30 (10%) |
| Capacity |  |  |  |  |  |  |  |  |  |  |
| I am able to do heart physical examination correctly | 0 (0) | 3 (1%) | 20 (8%) | 99 (40%) | 123 (50%) | 2 (0%) | 23 (7%) | 68 (23%) | 143 (49%) | 52 (18%) |
| Application |  |  |  |  |  |  |  |  |  |  |
| I am able to interpret the heart physical examination result | 0 (0) | 8 (3%) | 34 (13%) | 120 (48%) | 83 (33%) | 5 (1%) | 34 (11%) | 110 (38%) | 113 (39%) | 26 (9%) |
| Intended Behaviour |  |  |  |  |  |  |  |  |  |  |
| With my competence, I will do heart physical examination when i meet the patient | 0 (0) | 8 (3%) | 61 (24%) | 94 (38%) | 82 (33%) | 7 (2%) | 39 (13%) | 100 (34%) | 109 (37%) | 33 (11%) |
| Self-Efficacy |  |  |  |  |  |  |  |  |  |  |
| I able to achieve the heart physical examination competence according to learning objective | 0 (0) | 3 (1%) | 40 (16%) | 104 (42%) | 98 (0,4) | 2 (0%) | 28 (9%) | 106 (36%) | 123 (42%) | 29 (10%) |
| SATISFACTION | | | | | | | | | | |
| I satisfy with the guidance for instructor | 0 (0) | 1 (0%) | 16 (6%) | 81 (33%) | 147 (0,6) | 2 (0%) | 18 (6%) | 93 (32%) | 125 (43%) | 50 (17%) |
| More effort are needed for the preparation* | 76 (31%) | 86 (35%) | 34 (13%) | 25 (10%) | 24 (9%) | 8 (2%) | 23 (7%) | 47 (16%) | 99 (34%) | 111 (38%) |
| More effort are needed during the course* | 96 (39%) | 92 (37%) | 22 (8%) | 16 (6%) | 19 (7%) | 28 (9%) | 81 (28%) | 81 (28%) | 78 (27%) | 20 (6%) |
| I satisfy with the facility of the course | 1 (0%) | 3 (1%) | 27 (11%) | 85 (34%) | 129 (52%) | 11 (3%) | 29 (10%) | 102 (35%) | 103 (35%) | 43 (14%) |
| I satisfy with the learning objectives | 0 (0) | 0 (0) | 21 (8%) | 94 (38%) | 130 (53%) | 0 (0%) | 6 (2%) | 75 (26%) | 154 (53%) | 53 (18%) |
| I satisfy with the learning method of the course | 0 (0) | 2 (0%) | 19 (7%) | 97 (39%) | 127 (51%) | 14 (4%) | 33 (11%) | 120 (41%) | 99 (34%) | 22 (7%) |

# *considered as negative questions
